# COVID-19 infection at a psychiatric hospital in KwaZulu-Natal, South Africa: Clinical service planning and challenges

**DOI:** 10.4102/sajpsychiatry.v28i0.1933

**Published:** 2022-12-07

**Authors:** Saeeda Paruk, Ntokozo N. Ngcobo, Enver Karim, Andrew Tomita, Suvira Ramlall

**Affiliations:** 1 Department of Psychiatry, School of Clinical Medicine, University of KwaZulu-Natal, Durban, South Africa; 2 KwaZulu-Natal Research Innovation and Sequencing Platform (KRISP), College of Health Sciences, University of KwaZulu-Natal, Durban, South Africa; 3 Centre for Rural Health, School of Nursing and Public Health, University of KwaZulu-Natal, Durban, South Africa

**Keywords:** COVID-19, pandemic, psychiatric service, mental illness, South Africa

## Abstract

**Background:**

South Africa had over 4 million cases of coronavirus disease 2019 (COVID-19) infections and more than 1 million COVID-19-related deaths. Despite the devastating psychological impact of the COVID-19 pandemic, there is little qualitative, critical evaluation of government mental health services in this resource-limited setting.

**Aim:**

The authors describe the clinical service plan and response to the COVID-19 pandemic at a government psychiatric hospital.

**Setting:**

KwaZulu-Natal, South Africa.

**Methods:**

A descriptive narrative overview of the specialised psychiatric hospital’s clinical response (April 2020 – March 2021) to the COVID-19 pandemic was undertaken in the following domains: screening policy; testing and swabbing policy; staff training and monitoring; and restructuring the wards to accommodate mental health care users (MHCUs) with suspected cases of COVID-19.

**Results:**

The in-depth narrative reviews led to the introduction of staff training, routine COVID-19 reverse transcription polymerase chain reaction (RT-PCR) testing of all MHCUs, the creation of designated quarantine and isolation facilities and screening of physical health status of patients with COVID-19 prior to transfer being implemented to prevent an outbreak or increased morbidity or mortality.

**Conclusion:**

Implementing a service plan early which included staff training, screening and routine COVID-19 testing services for psychiatric admissions in a rapidly evolving environment with few additional resources was challenging. The absence of guidelines early in the pandemic that addressed the unique needs of a clinical psychiatric inpatient population is a noteworthy learning point.

**Contribution:**

The article highlights that the inpatient infrastructural requirements and clinical management protocols of acutely psychiatrically ill inpatients, in the context of infectious outbreaks, require dedicated task teams and bespoke policies.

## Introduction

The coronavirus disease 2019 (COVID-19) pandemic, which started with the first diagnosed case in December 2019 in China, has necessitated a global change in behaviour and continues to impact day-to-day functioning more than two years later.^1^ The first South African (SA) case was reported in March 2020, the country having the highest number of reported cases in Africa, possibly because of its better testing capacity.^1^ While much attention has been focused on the mental health impact of the pandemic, considerably less has been on its impact on patients with chronic, severe psychiatric disorders. Furthermore, national and international COVID-19 treatment guidelines for patients under investigation (PUIs) and those infected were silent on the unique challenges posed in quarantining, isolating, physical distancing and masking patients who were acutely psychiatrically disturbed in a limited resource setting early in the pandemic.^2,3^

Psychiatric hospitals have limited resources to screen and treat medical comorbidities and are seldom linked to a general medical health service. Mental health care facilities often have limited space and general resource constraints, with high inpatient numbers and patient turnover, which increases the risk for COVID-19 infections and its spread.^3^ Compared to general hospitals, patients in psychiatric hospitals are long term, share common dining and bathroom spaces, participate in group activities and interact closely, all of which increase patient-to-patient contact.^4,5^ These factors necessitated bespoke plans and management protocols for inpatient psychiatric units to optimise the safety of both staff and patients.^4^

It is arguable whether any hospital in the world was optimally structurally designed or clinically equipped to accommodate the demands of the COVID-19 pandemic. Except for seclusion rooms, inpatient psychiatric hospitals are generally not designed or intended to isolate patients, especially highly infectious ones, from each other. Therefore, while the current pandemic posed unique challenges for infection prevention and control within health care facilities in general,^6^ they were greater for acute psychiatric inpatient settings.^6^ According to a survey by the World Health Organization (WHO), the pandemic disrupted or delayed mental health services worldwide.^7^ Factors such as the lack of screening protocols and preventive measures to reduce the risk of infection, as well as limited resources, negatively impacted the quality of care.^7^ The outbreaks that occurred in psychiatric facilities during the first wave of the COVID-19 pandemic in 2020 highlighted the need for such facilities to provide a safe environment for mental health care users (MHCUs) and staff.^8^ The standard COVID-19 guidelines on infection prevention and control to guide general health institutions during the pandemic did not address the unique nature of mental health care facilities or the needs of MHCUs, with South Africa being no exception.^9^ This occurred despite considerable evidence that the psychiatric population is vulnerable to infection for several reasons.^10,11,12^

Recent data have confirmed that MHCUs are at an increased risk for COVID-19 infection compared to individuals without a mental illness.^12,13^ This may be because of the nature and severity of their illness, negative health-related behaviours, decreased ability to follow COVID-19 containment measures, medical comorbidities and suboptimal living environments.^3,14^ Mental health care users are often unable to give a reliable history of COVID-19 symptoms, highlighting the need for mandatory testing in psychiatric facilities to protect the other patients and staff and for clinical observations of the COVID-19 infection.^8^ Pre-existing mental disorders, severe mental illness, intellectual disability, substance use disorders and previous exposure to psychopharmacological compounds have been found to be associated with poor COVID-19 outcomes.^15^ As there has been little data on the COVID-19 experience within psychiatric facilities on the African continent, the authors aim to describe the clinical service response to the pandemic.

## Methods

This study was a retrospective review of the adaptation of an inpatient psychiatric service to meet the clinical guidelines for quarantining and isolating patients during the COVID-19 pandemic. The authors first describe the profile of inpatients who tested positive for COVID-19 during the first year of the pandemic (01 April 2020 to 01 March 2021) using a retrospective chart review, their clinical data having been captured by two psychiatrists. The authors then provide a descriptive narrative overview of the specialised psychiatric hospital’s clinical response to the COVID-19 pandemic in the following domains: screening policy; testing (COVID-19 swabbing) policy; staff training and monitoring; and restructuring the wards to accommodate MHCUs under investigation for COVID-19.

### Setting

The study was conducted at a specialist psychiatric hospital in the eThekwini district, KwaZulu-Natal (KZN) province, South Africa, that serves an extensive part of surrounding areas up to and including parts of the Eastern Cape province. It is one of only three public sector psychiatric hospitals in KZN, the province having a population of approximately 11 million people.^16^ The psychiatric unit consists of inpatient (currently 68 beds) and outpatient services and is part of an academic teaching complex for the province’s medical school.

### Study population

The study population comprised acutely mentally ill patients (14 years and older) admitted to the psychiatry hospital between 01 April 2020 and 01 March 2021 who tested COVID-19 positive on reverse transcription polymerase chain reaction (RT-PCR) tests.

### Measurements

A narrative review of the psychiatric unit’s COVID-19 clinical response is described based on the South African Department of Health’s COVID-19 policies and guidelines, the minutes of hospital meetings and the experiences of the authors at the psychiatric service for the study period.

A structured sociodemographic and clinical data sheet based on a review of the literature was used to collate data on the profiles of MHCUs admitted with COVID-19 infection in the first 11 months of the pandemic. Sociodemographic information included MHCUs’ race, age, gender, first language, highest level of education and place of residence. Clinical and treatment data collated included the provisional diagnosis of the patient according to the *Diagnostic and Statistical Manual of Mental Disorders, Fifth Edition* (DSM-5) criteria,^17^ comorbid psychiatric and medical disorders, including human immunodeficiency virus (HIV) status, Mental Health Care Act (MHCA)^18^ admission status, presenting systems, self-report of adherence to treatment prior to admission, haematological results and medication management.

### Data analysis

A descriptive analysis was undertaken of actions taken by the multidisciplinary staff (psychiatric, nursing and psychology services) at the unit and a critical evaluation of national and international practices against the adopted policies and practices at the studied facility. Where relevant, the rationale for the departure from the national or international policy or practice is described. The details of the planning process and policies formulated and implemented by the multidisciplinary mental health team are described for the first (July 2020 – September 2020) and second (December 2020 – February 2021) waves of the pandemic, as experienced in KZN. Given the small sample size, the descriptive statistics focused on nonparametric statistics using Stata version 17 (StataCorp LLC, College Station, Texas, United States), with no inferential statistics being considered. Data analysis involved the use of frequencies, percentages, median and interquartile ranges.

### Ethical considerations

Ethical approval was obtained from the University of KwaZulu-Natal Biomedical Research Ethics Committee (ref. no. BREC-00002679/2021), the hospital and the Department of Health.

## Results

### Sociodemographic of mental health care users admitted with COVID-19 infection

Mental health care users’ sociodemographic profiles are summarised in Table 1, and it shows that during the 11-month study period of the 207 MHCUs admitted, 19 tested COVID-19 RT-PCR positive for COVID-19 infection (*n* = 19). Fifteen tested positive following routine testing at admission, and four patients with confirmed COVID-19 were referred to the unit for containment, as they were too disruptive and could not be isolated in a medical ward for COVID-19 patients at the referring hospitals. The majority of MHCUs who tested COVID-19 RT-PCR positive were black males, and their ages ranged from 14 to 66 years (mean 29.7, standard deviation [s.d.] 13.8).

**TABLE 1:** Sociodemographic characteristics of mental health care users testing COVID-19 reverse transcription polymerase chain reaction positive (*N* = 19) from a total of 207 admissions.

Sociodemographics	Characteristics	Overall	
		*n*	%
Race	Black people	18	94.7
	Other	1	5.3
Employment status	Employed	1	5.3
	Unemployed	14	73.7
	On disability grant	4	21.1
Gender	Male	14	73.7
	Female	5	26.3
Home language	IsiZulu	18	94.7
	English	1	5.3
Level of education	Grade 1–7	4	21.1
	Grade 8–12	15	98.9
	Tertiary education	0	0.0
Residential area	uMgungundlovu	1	5.3
	eThekwini	17	89.5
	Other	1	5.3

### Clinical profile of mental health care users admitted with COVID-19 infection

All 19 MHCUs with COVID-19 RT-PCR positive tests presented with either a relapse (*n* = 15) or new onset (*n* = 4) of psychotic symptoms. The primary clinical psychiatric diagnoses in these 19 MHCUs were schizophrenia and schizophrenia spectrum disorders (*n* = 16, 84.2%), bipolar mood disorder (*n* = 2, 36.8%) and major neurocognitive disorder (*n* = 1). All received antipsychotics, with their other clinical variables being summarised in Table 2.

**TABLE 2:** Clinical characteristics of mental health care users testing COVID-19 reverse transcription polymerase chain reaction positive (*N* = 19).

Clinical characteristics	Variable	Overall	
		*n*	%
Mental Health Care Act status	Voluntary	1	5.3
	Assisted	0	0.0
	Involuntary	18	94.7
Past psychiatric history	Yes (relapse)	14	73.7
	No	5	26.3
Adherence to maintenance treatment	Yes	1	5.3
	No	18	94.7
Substance use	Yes	13	68.4
	No	6	31.6
Common presenting symptoms	Disorganised behaviour†	18	94.7
	Delusions†	18	94.7
	Hallucinations†	17	89.5
	Disorganised speech†	16	84.2
	Aggression†	16	84.2
	Insomnia†	7	36.8
	Elevated mood†	5	26.3
	Self-injurious behaviour	2	10.5
	Cognitive†	2	10.5
Comorbid medical illness	Yes	9	47.4
	No	10	52.6
Type of medical comorbidity	HIV†	6	31.6
	Hypertension†	5	26.3
	Epilepsy†	1	5.3
	Hypercholesterolaemia†	1	5.3
COVID-19 status known on admission	Yes	4	21.1
	No	15	78.9
Screening status (*n* = 15)	Negative	8	53.3
	Positive	7	46.7
Reason for screening positive	Presence of COVID-19 symptoms†	4	21.1
	COVID-19 case contact†	3	15.8
Reason for testing	Admission policy	13	68.4
	Screened positive	6	31.6
COVID-19 infection medical status in ward	Asymptomatic	13	68.4
	Respiratory symptoms	6	31.6
Presumed source of COVID-19 based on length of hospital stay at referral hospital before transfer	Hospital-acquired	12	63.2
	Community-acquired	7	36.8
Abnormal haematological investigations	Full blood count†,‡	5	26.3
	Urea and electrolytes (low sodium)†	1	5.3
	Liver function test – raised liver enzyme†	1	5.3
	Elevated erythrocyte sedimentation rate (ESR)†	9	47.4
	Elevated C-reactive protein (CRP)†	8	42.1

HIV, human immunodeficiency virus; COVID-19, coronavirus disease 2019.

† , Denominator is 19;

‡ , Full blood count: three had mild anaemia, one had raised white cells and one had decreased white cells.

Of the 19 MHCUs who were COVID-19 RT-PCR positive, 13 (68.4%) were asymptomatic throughout the isolation period and six (31.6%) had mild respiratory symptoms (cough or nasal congestion with no respiratory distress and not requiring oxygen). There were no deaths and no patient required high-care medical services. Clinicians ascribed seven (36.8%) COVID-19 infections to community acquisition and 12 (63.2%) as hospital-acquired, based on the duration of stay at the referral hospital prior to referral to the psychiatry hospital and being tested.

Haematological tests were conducted on all MHCUs testing COVID-19 RT-PCR positive, the erythrocyte sedimentation rate (ESR) being elevated in nine (47.4%) and the C-reactive protein (CRP) also being abnormally increased in 8 of the 19 (42.1%). Of the 17 MHCUs who had chest X-rays, only one (5.9%) had an atypical pneumonia and four (23.5%) had old infective changes.

### Main findings regarding clinical service response

As with the rest of the world, no hospital was structurally configured to meet the unique demands and magnitude of need that COVID-19 posed. The psychiatric hospital laboured under significant resource (infrastructural and human) constraints to fulfil service demands before the pandemic. While the National Department of Health (NDOH) responded to the pandemic by designating health care facilities to provide isolation and high-care treatment and establishing ‘field hospitals’, their policy was notably lacking in a plan for acutely psychiatrically ill individuals who contracted the virus.^19^ Guidelines were also lacking from the WHO and NDOH regarding a testing policy for MHCUs.^19,20^

The hospital is part of a health care complex that also houses a district-level nonspecialist general hospital. While the operations of the general hospital were guided by national and international policies, a policy or dedicated facility that addressed the unique needs of acutely mentally ill patients was lacking. The following COVID-19 infection risk mitigation strategies were thus developed as the pandemic evolved.

Firstly, the clinical staff at the study site acknowledged the inability of acutely mentally ill patients to be reliably screened for COVID-19 using the existing screening tools, which relied largely on self-report. A decision was therefore made to swab every MHCU on admission and quarantine them until their COVID-19 infection status was confirmed, before integrating them into the existing inpatient population. Because of the limited testing resources and uncertainty about the virus in the early stages of the pandemic, this decision met with resistance initially from referring hospitals and the hospital administration, as it was not regarded as ‘evidence-based’, informed by national policy or economically viable with respect to resources. However, in the interests of patient safety and protecting the vulnerable inpatient cohort, the practice was maintained and, as the pandemic progressed, in-principle support was received from the provincial mental health directorate, this practice being later adopted by other psychiatric inpatient units in the province.

Secondly, the requirement by the hospital to have ‘red’ (contaminated or COVID-19 positive), ‘orange’ (potentially contaminated or ‘person under investigation’) and ‘green’ (noninfectious) zones or clinical areas based on the COVID-19 status of ‘positive’ ‘PUI’ or ‘negative’ patients, respectively,^21^ posed a major challenge infrastructurally. Not only are the wards in the psychiatric hospital not designed to address the quarantine and isolation needs of MHCUs, but their layout proved cumbersome to maintain the designated zones and allow for the requisite donning and doffing areas. It required a creative use of space to ensure compliance with health and safety regulations, but it ultimately resulted in a reduction of admission capacity, which was exacerbated by the need to separate acutely mentally ill male and female patients, thereby requiring two quarantine and isolation units each. The hospital initially rezoned one male and one female ward to allow for male and female red and yellow zones, respectively, which cut the total bed capacity by almost 50%; these beds were not optimally used because of the low numbers of COVID-19-infected patients at the time. This resulted in longer waiting periods for inpatients awaiting transfer into the hospital.

Thirdly, because of the low numbers of COVID-19-positive MHCU admissions, the hospital then compromised on separating patients by gender and cohabited male and female patients in a single wing of the ward in separate dormitories. This allowed the hospital to improve the utilisation of beds, as there were now 11 beds dedicated to PUI or COVID-19-infected individuals. However, for PUIs, there were considerable challenges in maintaining COVID-19-related infection risk mitigation strategies. Although available, confining acutely ill patients in separate dormitories proved unsuccessful, despite the optimal use of psychotropic medication and vigilance in nursing care. As numbers fluctuated between and during the surges, the four wards for PUIs and COVID-19-infected patients were accordingly redesignated or rezoned in order to ensure optimal management of these patients without unduly reducing the total capacity of the hospital to accommodate non-COVID-19 patients. It must also be noted that despite the best efforts of staff, getting acutely psychotic patients to wear their masks and to remain in their own dormitories was a largely fruitless exercise.

Fourthly, as this psychiatry hospital is a referral hospital for other psychiatric units in the province, the limited capacity of the PUI wards resulted in longer delays than normal for awaiting transfers into the hospital. This was because of the waiting time for a COVID-19 RT-PCR result, which varied from eight hours to five days, based on the status of the pandemic in the country and the challenges faced by local laboratories. As KZN does not, to date, have designated isolation facilities for acutely mentally ill COVID-19-positive patients, outbreaks in other psychiatric units also necessitated transfers of confirmed COVID-19-positive MHCUs to this hospital to access the isolation wards.

The fifth (potential) challenge that was faced was the management of acutely mentally ill patients who were medically unstable and may have required ventilatory support. While the COVID-19 wards had the basic resuscitation and ambulant artificial ventilation support resources, the hospital was ill-equipped to manage respiratory distress beyond ‘stabilise and transfer’. Intensive care unit (ICU) beds during the surges were not easily available, if at all. To date, the hospital has not had a patient who required more than symptomatic treatment of mild flu symptoms, high care or ICU care because of the screening measures used prior to transferring the COVID-19 infected patients, such as baseline haematological tests, chest radio-imaging and physical examination. It is noteworthy that in August 2020, the provincial government did embark upon modifying an existing, unused building to serve as a high-care facility for MHCUs; it was completed in the latter part of 2021, but it was not commissioned for a variety of reasons.

Finally, frequent education and training of all staff on the screening, swabbing, monitoring and management protocols were conducted. This proved challenging, as the guidelines and policies were not always available as they were still being formulated, and there was no guiding policy that addressed the needs of infected, acutely mentally ill patients. While the training staff endeavoured to acquire and understand the policies, training the different cadres of staff (professional and nonprofessional, such as cleaners and security personnel), many of whom worked on different shifts, was challenging. Some of the staff struggled to appreciate the significance of respecting the contaminated and noncontaminated clinical area boundaries; others were confused about the use of personal protective equipment and felt anxiety about contracting the illness, these being parallel demands that had to be continually addressed while managing the infection-related needs of the inpatients.

## Discussion

In this study, the authors described the key measures undertaken to manage the COVID-19 infection risk in a psychiatric hospital and the profile of acutely psychiatrically ill MHCUs admitted with infection. The key findings were that 9% of MHCUs who were admitted in an 11-month period during the first and second waves tested COVID-19 positive, the majority of whom were asymptomatic and only detected on routine viral testing. Patients who tested positive were predominantly male, with a first language of isiZulu, and they presented with psychosis, having a pre-existing psychiatric diagnosis and a history of poor treatment adherence. While almost 50% of the 19 had comorbid medical disorders, with almost one third (31.6%, *n* = 6) of those testing positive for COVID-19 having comorbid HIV, all had mild disease and recovered from COVID-19. In addition, staff training, ward rezoning to accommodate PUIs and COVID-19-positive patients and testing all MHCUs on admission were critical to maintaining a service and limiting morbidity and mortality because of the COVID-19 infection.

With limited comparable data available, the prevalence of COVID-19 infection in this study suggests that patients with acute, severe mental illness are vulnerable to COVID-19 infection but may not be easily recognised. Arguably, the majority of those diagnosed in this sample would have evaded detection had they not been admitted for their psychiatric relapses. The possible role of the COVID-19 infection in precipitating a relapse, or exacerbating its severity, is a consideration. This may be partly explained by the inability of MHCUs to adhere to COVID-19 mitigation measures, which is supported in the literature.^5^ A study in the United Kingdom from 01 March to 30 April 2020 found that, of 344 inpatients, 131 (38%) with a mean age of 75.3 years were diagnosed with COVID-19 during the study period.^18^ The present study’s lower prevalence of COVID-19 infection is probably because of a much younger age group of MHCUs and the majority having short-term admissions for acute symptom relapse rather than long-term patients with neurocognitive disorders. The high rate supports the need for routine COVID-19 testing in people with acute, severe mental illness, even in the absence of COVID-19 symptoms, who may have difficulty adhering to COVID-19-related mitigation measures. This may be a useful strategy to avoid outbreaks in psychiatry wards.

Statistics on COVID-19 infection rates in SA found that women were more vulnerable,^14^ which was not borne out in this study, which may be because of a referral bias in the sample, as male patients tend to be more aggressive and less containable in nonspecialised psychiatric units. It should also be noted that many men and women living with mental illness may not have been referred to psychiatry services during this period, as health care services were remodelled to focus on those with severe COVID-19 infection, and families and MHCUs were reluctant to attend health care facilities for fear of contracting COVID,^19^ thus limiting the study findings. Clinicians ascribed 12 (63.2%) COVID-19 infections as hospital-acquired based on those patients not having had a positive contact or symptoms prior to their admissions to general hospitals, where they had spent over 10 days prior to transfer to the psychiatric hospital. This suggests that many public general hospitals with under-resourced facilities did not have the capacity to test and physically separate patients optimally.

The literature also suggests increased risk of death from COVID-19 for patients with mental illness because of challenges in health care access, medical comorbidities and/or lifestyle factors.^19^ The present study had no mortalities during the study period, which may be because of a referral bias, as it is less likely for severely physically ill patients to be simultaneously psychiatrically disruptive. The limited sample size and the selected population having been screened before admission limits the authors’ ability to comment on the impact of comorbid disorders, such as HIV and tuberculosis, which need to be explored in larger samples. The early implementation of an active COVID-19 training, ward rezoning, screening and testing policy may have also contributed to the lower morbidity and mortality at the facility.

### Limitations

This study is based on one clinical hospital site and hence introduces a sample bias and limits generalisability to community samples. However, it does provide a snapshot of the clinical service response and a sample representing the clinical profile of acutely psychotic patients with COVID-19 infection treated at a specialised psychiatry hospital. The retrospective review is limited by the quality of record-keeping, which may not be consistent or complete. Because of the cross-sectional nature of the study, longitudinal changes may have been missed, and the limited sample size did not allow for inferential statistical analysis. The possible long-term sequelae of COVID-19 on the medical and or psychiatric course of these patients are also not reportable, as the study site is a referral hospital.

## Conclusion

The pandemic has posed significant challenges to all clinical services, with policies and protocols evolving as information became available about the behaviour and clinical effects of the virus. Nonetheless, the conspicuous absence of guidelines early in the pandemic that addressed the unique needs of psychiatric clinical populations is a noteworthy learning point. Infrastructural and clinical management needs of acutely psychiatrically ill patients in the context of infectious outbreaks require dedicated task teams in order to uphold the rights of a vulnerable, marginalised and voiceless patient population.

## References

[1] World Health Organization. Coronavirus disease 2019 (COVID-19): Situation report 30. Geneva: World Health Organization; 2020.

[2] Kuzman MR, Vahip S, Fiorillo J, et al. Mental health services during the first wave of the COVID-19 pandemic in Europe: Results from the EPA ambassadors survey and implications for clinical practice. Eur Psychiatry. 2021;64(1):e41. 10.1192/j.eurpsy.2021.2215 34103102 PMC8314055

[3] Xiang YT, Zhao YJ, Liu ZH, et al. The COVID-19 outbreak and psychiatric hospitals in China: Managing challenges through mental health service reform. Int J Biol Sci. 2020;16(10):1741–1744. 10.7150/ijbs.45072 32226293 PMC7098035

[4] Chen J, Xiong M, He Z, Shi W, Yue Y, He M. The enclosed ward management strategies in psychiatric hospitals during COVID-19 outbreak. Global Health. 2020;16(1):1–2. 10.1186/s12992-020-00586-z 32580774 PMC7312105

[5] Rovers J, Van de Linde LS, Kenters N, et al. Why psychiatry is different-challenges and difficulties in managing a nosocomial outbreak of coronavirus disease (COVID-19) in hospital care. Antimicrob Resist Infect Control. 2020;9(1):1–8. 10.1186/s13756-020-00853-z 33261660 PMC7705849

[6] Rickman HM, Rampling T, Shaw K, et al. Nosocomial transmission of coronavirus disease 2019: A retrospective study of 66 hospital-acquired cases in a London teaching hospital. Clin Infect Dis. 2021;72(4):690–693. 10.1093/cid/ciaa816 32562422 PMC7337682

[7] Montes JM, Hernández-Huerta D. Impact of the COVID-19 pandemic on acute inpatient psychiatric units in Spain. Psychiatry Res. 2021;304:114136. 10.1016/j.psychres.2021.114136 34332433 PMC8310392

[8] Brody BD, Shi Z, Shaffer C, et al. COVID-19 infection rates in patients referred for psychiatric admission during a regional surge: The case for universal testing. Psychiatry Res. 2021;298:113833. 10.1016/j.psychres.2021.113833 33657449 PMC7901369

[9] Chun JY, Jun JY, Choi J, et al. Coronavirus disease 2019 outbreak in a psychiatric closed ward: What we have to learn. Front Psychiatry. 2021;11:579235. 10.3389/fpsyt.2020.579235 33551861 PMC7862577

[10] National Institute for Communicable Diseases. COVID-19 [homepage on the Internet]. 2021 [cited 2021 Sep 24]. Available from: https://www.nicd.ac.za/latest-confirmed-cases-of-covid-19-in-south-africa-31-mar-2021/

[11] Druss BG. Addressing the COVID-19 pandemic in populations with serious mental illness. JAMA Psychiatry. 2020;77(9):891–892. 10.1001/jamapsychiatry.2020.0894 32242888

[12] Taquet M, Luciano S, Geddes JR, Harrison PJ. Bidirectional associations between COVID-19 and psychiatric disorder: Retrospective cohort studies of 62 354 COVID-19 cases in the USA. Lancet Psychiatry. 2021;8(2):130–140. 10.1016/S2215-0366(20)30462-4 33181098 PMC7820108

[13] Wang Q, Xu R, Volkow ND. Increased risk of COVID-19 infection and mortality in people with mental disorders: Analysis from electronic health records in the United States. World Psychiatry. 2021;20(1):124–130. 10.1002/wps.20806 33026219 PMC7675495

[14] Shinn AK, Viron M. Perspectives on the COVID-19 pandemic and individuals with serious mental illness. J Clin Psychiatry. 2020;81(3):14205. 10.4088/jcp.20com13412 32369691

[15] Vai B, Mazza MG, Colli CD, et al. Mental disorders and risk of COVID-19-related mortality, hospitalisation, and intensive care unit admission: A systematic review and meta-analysis. Lancet Psychiatry. 2021;8(9):797–812. 10.1016/S2215-0366(21)00232-7 34274033 PMC8285121

[16] Stats SA Library Cataloguing-in-Publication (CIP) data. COVID-19 pandemic in South Africa, demography volume. Pretoria: Statistics South Africa; 2020.

[17] American Psychiatric Association. Diagnostic and statistical manual of mental disorders. 5th ed. Washington, DC: American Psychiatric Associate Publishing; 2013.

[18] Mental Health Care Act 2002 (Act no. 17 of 2002) General Regulations. Government Gazette, Republic of South Africa [homepage on the Internet]. c2004 [cited 2021 Oct]. Available from: http://www.info.gov.za/gazette/acts/2002

[19] Livingston G, Rostamipour H, Gallagher P, et al. Prevalence, management, and outcomes of SARS-CoV-2 infections in older people and those with dementia in mental health wards in London, UK: A retrospective observational study. The lancet. Psychiatry. 2020;7(12):1054–1063. 10.1016/S2215-0366(20)30434-X 33031760 PMC7535621

[20] World Health Organization. [homepage on the Internet]. No date [cited 2021 Nov]. Available from: https://www.nicd.ac.za/wp-content/uploads/2020/05/Guidelines-for-Quarantine-and-Isolation-in-relation-to-COVID-19.pdf

[21] Republic of South Africa Department of Health. Guidelines for quarantine and isolation in relation to COVID-19 exposure and infection. 2020 [cited 2021 Nov]. Available from: https://www.nicd.ac.za/wp-content/uploads/2020/05/Guidelines-for-Quarantine-and-Isolation-in-relation-to-COVID-19.pdf

